# Anti-Glucosylsphingosine Autoimmunity, JAK2V617F-Dependent Interleukin-1β and JAK2V617F-Independent Cytokines in Myeloproliferative Neoplasms

**DOI:** 10.3390/cancers12092446

**Published:** 2020-08-28

**Authors:** Sophie Allain-Maillet, Adrien Bosseboeuf, Nicolas Mennesson, Mégane Bostoën, Laura Dufeu, Eun Ho Choi, Cédric Cleyrat, Olivier Mansier, Eric Lippert, Yannick Le Bris, Jean-Marc Gombert, François Girodon, Magali Pettazzoni, Edith Bigot-Corbel, Sylvie Hermouet

**Affiliations:** 1 Institut National de la Santé et de la Recherche Médicale (INSERM), UMR 1232, CRCINA, University of Nantes, Institut de Recherche en Santé 2 (IRS-2), 22 Boulevard Benoni Goullin, 44200 Nantes, France; sophie.allain@inserm.fr (S.A.-M.); adrien.bosseboeuf@gmail.com (A.B.); nicolas.mennesson@univ-nantes.fr (N.M.); megane.bostoen@laposte.net (M.B.); dufeu.lau@gmail.com (L.D.); Yannick.LEBRIS@chu-nantes.fr (Y.L.B.); edith.bigot@univ-nantes.fr (E.B.-C.); 2 Department of Pathology & Comprehensive Cancer Center, University of New Mexico (NM) Health Sciences Center, Albuquerque, NM 87102 USA; ehchoi@salud.unm.edu (E.H.C.); cedric@cleyrat.com (C.C.); 3 Laboratoire d’Hématologie, CHU de Bordeaux, 33600 Pessac, France; olivier.mansier@gmail.com; 4 INSERM U1034, Université de Bordeaux, UFR Sciences de la Vie et de la Santé, 33000 Bordeaux, France; 5 Laboratoire d’Hématologie, CHU de Brest, 29200 Brest, France; eric.lippert@chu-brest.fr; 6 INSERM, Etablissement Français du Sang (EFS), UMR 1078, GGB, Université de Brest, 29200 Brest, France; 7 Laboratoire d’Hématologie, CHU de Nantes, 44093 Nantes, France; 8 Laboratoire d’Immunologie, CHU de Poitiers, 87000 Poitiers, France; Jean-Marc.GOMBERT@chu-poitiers.fr; 9 Laboratoire d’Hématologie, CHU Dijon, 21034 Dijon, France; francois.girodon@chu-dijon.fr; 10 INSERM, UMR 1231, University of Bourgogne Franche-Comté, 21078 Dijon, France; 11 LBMMS, Service de Biochimie et Biologie Moléculaire Grand Est, UF des Maladies Héréditaires du Métabolisme, Hospices Civils de Lyon, 69677 Bron CEDEX, France; magali.pettazzoni@chu-lyon.fr; 12 Laboratoire de Biochimie, CHU de Nantes, 44093 Nantes, France

**Keywords:** myeloproliferative neoplasms (MPNs), inflammation, cytokines, *JAK2*V617F, *CALR* exon 9 mutants, interleukin-1β (IL-1β), IL-1Rα, IP-10, leptin, IL-33, UT-7, CRISPR technology, antigenic stimulation, glucolipids, glucosylsphingosine (GlcSph), auto-immunity

## Abstract

**Simple Summary:**

Inflammation plays a major role in myeloproliferative neoplasms (MPNs) as regulator of malignant cell growth and mediator of clinical symptoms. Yet chronic inflammation may also be an early event that facilitates the development of MPNs. Here we analysed 42 inflammatory cytokines and report that in patients as well as in UT-7 cell lines, interleukin-1β and interferon-induced protein 10 (IP-10) were the main inflammatory molecules found to be induced by *JAK2*V617F, the most frequent driving mutation in MPNs. All other inflammatory cytokines were not linked to *JAK2*V617F, which implies that inflammation likely precedes MPN development at least in subsets of MPN patients. Consistently, a possible cause of early, chronic inflammation may be auto-immunity against glucolipids: we report that 20% of MPN patients presented with anti-glucosylsphingoside auto-antibodies. Since existing treatments can reduce glucosylsphingoside, this lysosphingolipid could become a new therapeutic target for subsets of MPN patients, in addition to *JAK2*V617F and inflammation.

**Abstract:**

Inflammatory cytokines play a major role in myeloproliferative neoplasms (MPNs) as regulators of the MPN clone and as mediators of clinical symptoms and complications. Firstly, we investigated the effect of *JAK2*V617F on 42 molecules linked to inflammation. For *JAK2*V617F-mutated patients, the *JAK2*V617F allele burden (%*JAK2*V617F) correlated with the levels of IL-1β, IL-1Rα, IP-10 and leptin in polycythemia vera (PV), and with IL-33 in ET; for all other molecules, no correlation was found. Cytokine production was also studied in the human megakaryocytic cell line UT-7. Wild-type UT-7 cells secreted 27/42 cytokines measured. UT-7 clones expressing 50% or 75% *JAK2*V617F were generated, in which the production of IL-1β, IP-10 and RANTES was increased; other cytokines were not affected. Secondly, we searched for causes of chronic inflammation in MPNs other than driver mutations. Since antigen-driven selection is increasingly implicated in the pathogenesis of blood malignancies, we investigated whether proinflammatory glucosylsphingosine (GlcSph) may play a role in MPNs. We report that 20% (15/75) of MPN patients presented with anti-GlcSph IgGs, distinguished by elevated levels of 11 cytokines. In summary, only IL-1β and IP-10 were linked to *JAK2*V617F both in patients and in UT-7 cells; other inflammation-linked cytokines in excess in MPNs were not. For subsets of MPN patients, a possible cause of inflammation may be auto-immunity against glucolipids.

## 1. Introduction

An active JAK2/STAT5 pathway is required for an appropriate production of mature myeloid cells. Strong and prolonged activation of the JAK2/STAT5 pathway by erythropoietin (EPO), thrombopoietin (TPO), granulocyte-colony stimulating factor (G-CSF) or certain interleukins, physiologically enhances myelopoiesis, for instance after severe bleeding or during acute or chronic inflammation. The different chronic myeloproliferative neoplasms (MPNs) typically arise from the acquisition in a multipotent hematopoietic progenitor of one mutation in *JAK2*, *CALR* or *MPL*, and the subsequent mutant protein stimulates the expansion of mutated myeloid cells via constant activation of the JAK2/STAT5 pathway [1,2,3,4,5,6,7,8]. Hence MPNs represent clonal versions of myelopoiesis. However, *JAK2* mutation may occur more than once in certain patients and is not always the first event in MPNs [9,10]. Moreover, MPNs are associated with chronic inflammation, per se a strong stimulant of myelopoiesis. In MPNs, TPO and EPO levels are low or undetectable, but MPN patients have high blood levels of numerous inflammatory cytokines; some of these cytokines activate JAK2/STAT5 (G-CSF, granulocyte-macrophage colony stimulating factor (GM-CSF), interleukin 6 (IL-6)) while others activate the JAK1/STAT1/STAT3 pathways, notably IL-6 and interferons (IFN) [11,12].

Three subtypes of MPNs are distinguished: essential thrombocythemia (ET), which concerns mostly megakaryocytes and platelets; polycythemia vera (PV), which concerns predominantly the erythroid lineage; and primary myelofibrosis (PMF), a subtype characterized by severe fibrosis of the bone marrow and splenomegaly. The *JAK2*V617F mutation is found in >95% PV cases and 50–60% of ET and PMF cases, while *CALR* mutations characterize 25–30% ET and PMF cases; *MPL* mutations concern 5–10% ET and PMF cases. Patients may present with clinical symptoms and complications that include fatigue, fever, night sweats, loss of weight, itching, arterial and venous thrombosis, bone marrow fibrosis and splenomegaly; evolution toward acute myeloid leukemia (AML) is rare [13]. Most of these symptoms and complications, including bone marrow fibrosis, can be explained by inflammation. Logically, JAK inhibitors that significantly reduce inflammation also reduce clinical symptoms and splenomegaly [14,15,16,17,18,19]. Unfortunately, suppression of the MPN clone and significant reduction in the mutation load are typically not obtained with JAK inhibitors [17,18,19]. In contrast, IFN-α therapy frequently leads to clinical and molecular remission, in PV and also in *JAK2*- and *CALR*-mutated ET [20,21,22,23]. One explanation is that the actions exerted by JAK inhibitors and IFN-α on inflammation are quite different. JAK inhibitors block the myelopoiesis-stimulating JAK2/STAT5 pathway and often also the inflammation-linked JAK1/STAT1 pathways. In contrast, IFN-α is a potent immunostimulant that activates the JAK1/STAT1 pathways, thus inducing the expression of pro-inflammatory cytokines: IFN-induced protein 10 (IP-10), IL-6, IL-8, IL-10, GM-CSF and tumor necrosis factor α (TNF-α). However, IFN-α also represses the expression of cytokines and receptors that do not signal via JAK/STAT, notably IL-1β, a major pro-inflammatory cytokine, as well as others that facilitate the survival of MPN progenitors, such as IL-11, hepatocyte growth factor (HGF) and its receptor, c-MET, and tumor growth factors β (TGF-β) [24,25]. Consistently, IFN-α and JAK inhibitors were reported to act in synergy in MPNs [26,27,28]. Hence, both chronic inflammation and the *JAK2*/*CALR*/*MPL* mutants play major roles in the pathogenesis of MPNs, and inflammation cytokines act as stimulants of the mutated clone as well as mediators of clinical symptoms and complications [12,29].

Yet the mechanisms and chronology of inflammation in MPNs remain poorly understood [30]. Still debated are the causes of the excessive production of cytokines in MPNs (mutations, others?), and whether inflammation may precede the acquisition of mutations in *JAK2*/*CALR*/*MPL* genes. Several studies reported that a history of chronic inflammation of various causes (smoking, auto-immune disease, inflammatory rheumatisms, inflammatory bowel disease) is not rare in patients who develop an MPN [30,31,32,33]. Importantly, nongenetic pathogenic mechanisms such as chronic antigen stimulation and antigen-driven selection are increasingly implicated in the pathogenesis of blood malignancies. In the context of monoclonal gammopathies of undetermined significance (MGUS) and myeloma, Nair et al. and our own studies showed that the monoclonal immunoglobulins (Igs) of subsets of patients react against a pro-inflammatory lysosphingolipid, glucosylsphingosine (GlcSph) [34,35,36,37,38]. Furthermore, up to 40% of MGUS and myeloma patients had polyclonal anti-GlcSph antibodies, which implied that an auto-immune process accompanied MGUS or myeloma disease in these patients [38]. GlcSph—also called lysoglucosylceramide (LGL1)—is a frequent target of monoclonal Igs in MGUS and myeloma associated with Gaucher disease (GD) [34,35,36]. In GD, germline mutations in the glucocerebrosidase (*GBA*) gene result in the accumulation of glucocerebroside corresponding to the primary accumulated sphingolipid, and its deacylated form, the GlcSph. GD patients may present with various clinical manifestations, including an increased risk of developing blood malignancies, notably MGUS and myeloma [39,40,41,42,43].

In this study, our first aim was to determine the effect of *JAK2*V617F expression on 40 cytokines and 2 cytokine receptors, both in vivo (MPN patients) and in vitro (human UT-7 cells genetically engineered to express MPN mutations). We report that among 26 cytokines found to be overexpressed in *JAK2*V617F-mutated MPN patients, 23 could not be linked to *JAK2*V617F expression. Consequently, our second aim was to search for causes of chronic inflammation in MPNs other than mutation(s), and we investigated whether GlcSph stimulation played a role in MPNs.

## 2. Results

### 2.1. Description of Patients

In this retrospective study, serum samples collected from 75 patients at the time of diagnosis of MPN (27 PV, 39 ET, 9 PMF) and 54 healthy donors (HDs) (including 40 individuals of age >60) were aliquoted, then kept frozen at −80 °C. In the MPN cohort, the usual representation of *JAK2*V617F, *CALR* and *MPL* mutants was respected since it included 55/75 (73.3%) patients with a *JAK2*V617F-mutated MPN (27 PV, 21 ET, 7 PMF), 16/75 (21.3%) patients with a *CALR*-mutated MPN (15 ET, 1 PMF) and 2 ET patients with an *MPL* mutation (2/75 or 2.7%). Table 1 shows the characteristics of the MPN cohort. The median age of MPN patients at the time of diagnosis was 69 years for PV, 73 years for ET and 62 years for PMF. For *JAK2*V617F-mutated MPN patients, the percentage of *JAK2*V617F-mutated alleles as assessed in genomic DNA (gDNA) ranged from 1% to 100% (Supplementary Figure S1). We and others previously showed that the %*JAK2*V617F in mRNA and gDNA are well correlated [44]. Thus, the expression level of the *JAK2*V617F mutant in this cohort of patients presumably ranged from 1% to 100% of total JAK2. The percentage of *CALR*-mutated alleles in gDNA was close to 50% for all *CALR*-mutated patients.

### 2.2. Levels of Inflammation-Linked Cytokines in MPN Patients

Forty cytokines and chemokines and two receptors linked to inflammation were measured in the blood serum of 75 MPN patients and 17 HDs using the Luminex technology and Bio-Plex Pro human cytokine panel kits, as previously described [45]. We found significantly high levels for 26 cytokines (Table 2, indicated in blue) in MPN patients, compared to HDs (*p* < 0.05, Mann–Whitney *t*-test). Of note, 11 of the 26 cytokines are produced by cell types which are typically not mutated in MPNs, such as stromal cells (IL-7, basic fibroblast growth factor (b-FGF)), stroma-derived factor 1α (SDF-1α), endothelial cells (G-CSF, vascular endothelial growth factor (VEGF)), activated T-cells (IL-2, IL-2Rα, IL-5, IL-4, IL-9, IL-17, IFN-γ). The other cytokines overexpressed in MPNs are produced by potentially mutated CD34+ progenitor cells (HGF, MIP-1β, IL-33, IFN-α_2_) or monocytes–macrophages (11 cytokines, including IL-1β). Intriguingly, since most patients had high neutrophil counts, JAK2-activating cytokines were also elevated, notably G-CSF, IL-6, IL-5 and IFN-γ. Other JAK/STAT activators elevated in MPNs included activators of JAK1 (IFN-α_2_, IFN-γ, IL-6, IL-5, IL-26), JAK1/3 (IL-2, IL-7, IL-9, IL-15) and JAK3/STAT6 (IL-4).

### 2.3. Differences in Inflammation-Linked Cytokine Levels According to MPN Phenotype

**Figure 1:**
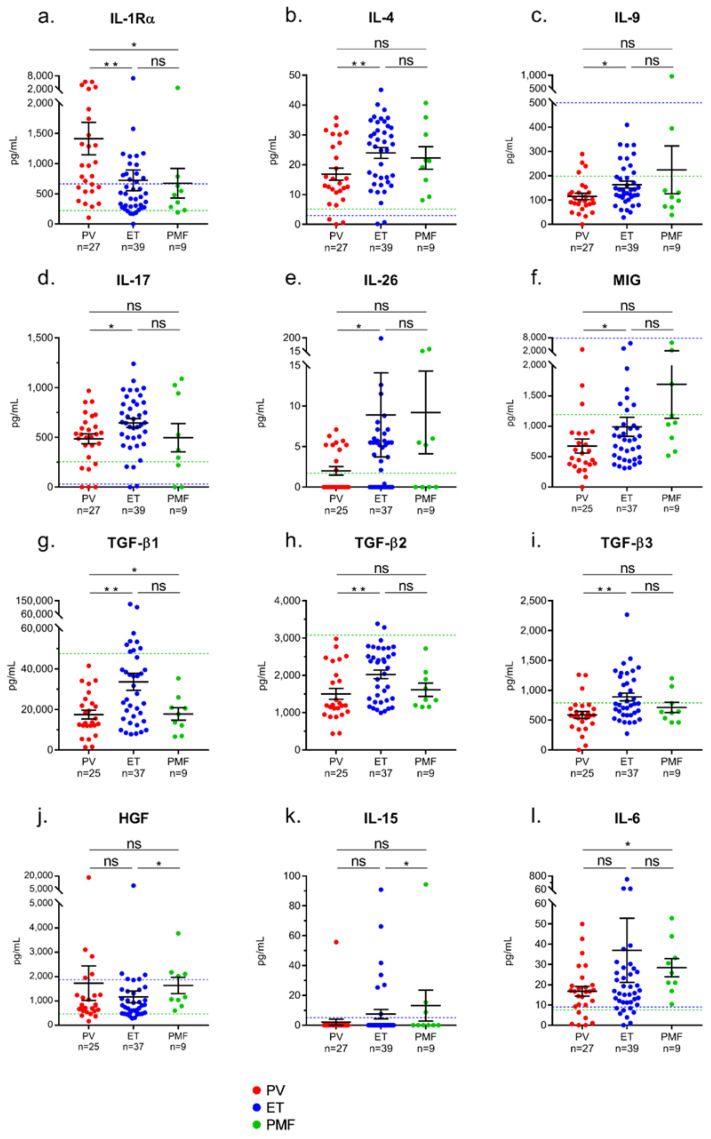
Differences in cytokine levels between PV, ET and primary myelofibrosis (PMF). Significant differences were found between PV, ET or PMF patients in the levels of 12 cytokines: (**a**) IL-1Rα; (**b**) IL-4; (**c**) IL-9; (**d**) IL-17; (**e**) IL-26; (**f**) MIG; (**g**) TGF-β_1_; (**h**) TGF-β_2_; (**i**) TGF-β_3_; (**j**) HGF; (**k**) IL-15; (**l**) IL-6. Results are presented as the means + SEM. NS: not significant. (*) *p* < 0.05 and (**) *p* < 0.01, Mann–Whitney *t*-test. Dotted blue lines represent the upper normal values for healthy individuals according to the manufacturers of the BioPlex Pro-human Cytokine kits, measured in 66 healthy donors. Dotted green lines represent the upper normal values for healthy individuals measured in the 17 healthy donors of our control cohort.

We compared the levels of cytokines observed for PV patients to those of ET and PMF patients. PV patients differed from ET and PMF patients by a high level of IL-1Rα (median: 966 ng/mL for PV vs. 501 ng/mL for ET and 480 ng/mL for PMF, *p* = 0.002 and *p* = 0.032, respectively; Mann–Whitney *t*-test) (Figure 1a, Table 2). Compared to PV, ET was characterized by high levels of four T-cell-derived interleukins (IL-4, IL-9, IL-17, IL-26) and four cytokines produced by monocytes–macrophages: monokine induced by IFN-γ (MIG), TGF-β_1_, TGF-β_2_ and TGF-β_3_ (Figure 1b–h, Table 2). PMF was characterized by high levels of HGF, IL-15, IL-6, and MIG (Figure 1i–l, Table 2). The levels of IL-1β, IP-10 and IL-33 and leptin were similar in PV, ET and PMF (Table 2). The levels of soluble IL-33 receptors (ST-2) were also examined, both in serum and in plasma. Supplementary Figure S2 shows that plasma ST-2 levels were significantly higher for PV, ET and PMF patients than for healthy controls, but there was no significant difference depending on MPN phenotype.

### 2.4. Correlations between Cytokine Levels and Blood Cell Counts in MPN Patients

We and others reported that when PV and ET patients are studied separately, the %*JAK2*V617F correlates with the leukocyte and neutrophil counts but not with the hematocrit or hemoglobin level, nor with platelet counts [44,46]. These observations were also true for the present cohort of patients (see Supplementary Table S1). We hypothesized that blood parameters of patients might be linked to the level of particular cytokines. Indeed, positive correlations were found between the levels of 13 molecules (8 cytokines, 3 chemokines, 2 receptors) and blood counts of neutrophils (correlated well in PV with IL-1Rα, and weakly with IL-1β, IP-10, HGF and MIG), monocytes (weak correlation with IP-10 in PV), lymphocytes (weak correlations with IL-4, IL-9, IL-33 and MIP-1β) and platelets (weak correlations with SDF-1α, IL-2Rα, IL-7, IL-9, IL-17 and MIG) (Table 3).

Inverse correlations were also observed between the levels of 8 molecules (leptin, 5 cytokines, 2 chemokines) and blood counts of neutrophils (negative correlation with leptin in PV), monocytes (negative correlation with leptin, IL-8 and MIP-1β in PMF), lymphocytes (negative correlation with MIP-1α and MIP-1β in PMF) and platelets (negative correlation with TGF-β_1_ in ET) (Table 3). No correlation was found between cytokine levels and hematocrit or hemoglobin in PV and in PMF, whereas in ET, weak inverse correlations were found between hematocrit and IL-2, IL-4 and IL-26 (Supplementary Figure S3).

### 2.5. Inflammation-Linked Cytokines or Receptors Linked to the JAK2V617F Mutation

**Figure 2:**
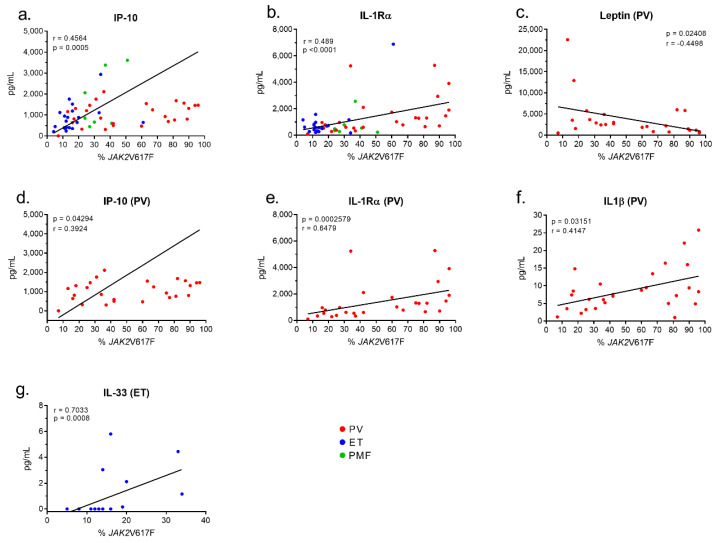
Correlations between cytokine levels and % of *JAK2*V617F-mutated alleles. Analysis of the cytokine levels and %*JAK2*V617F of the 55 patients with *JAK2*V617F-mutated MPN, using Spearman’s *t*-test, revealed positive correlations between %*JAK2*V617F and IP-10 (**a**) and IL-1Rα (**b**), and a negative correlation between leptin and %*JAK2*V617F, in PV only (**c**). (**d**–**f**) Positive correlations in PV only between the %*JAK2*V617F and IP-10 (**d**), IL-1Rα (**e**) and IL-1β (**f**). (**g**) Positive correlation between IL-33 and %*JAK2*V617F in ET only.

In the cohort of 55 patients with *JAK2*V617F-mutated MPN (27 PV, 21 ET, 7 PMF), the levels of cytokines in blood serum were analyzed according to the %*JAK2*V617F. Among the 42 molecules quantified, only macrophage-produced IP-10 and the receptor IL-1Rα correlated positively (weakly) with the %*JAK2*V617F of patients: IP-10: *n* = 55, *r* = 0.456, *p* = 0.0005; IL-1Rα: *n* = 55, *r* = 0.489, *p* = 0.0002 (Figure 2a,b). For PV patients, the %*JAK2*V617F and the blood level of leptin were inversely correlated, particularly for male patients (all patients with PV: *n* = 27, *r* = −0.450, *p* = 0.0241; men with PV, *n* = 16, *r* = −0.714, *p* = 0.0026) (Figure 2c). Weak positive correlations between the %*JAK2*V617F and levels of IP-10 and IL-1Rα were confirmed in the PV cohort (IP-10: *n* = 27, *r* = 0.392, *p* = 0.0429; IL-1Rα: *n* = 27, *r* = 0.648, *p* = 0.0003) (Figure 2d,e). In addition, the %*JAK2*V617F and IL-1β were correlated (*n* = 27, *r* = 0.415, *p* = 0.0315) for PV patients (Figure 2f). In contrast, in the cohort of *JAK2*V617F-mutated ET, IL-33 was the only cytokine correlated with the %*JAK2*V617F (*n* = 21, *r* = 0.703, *p* = 0.008) (Figure 2g).

Hence, the level of expression of the *JAK2*V617F mutant correlated with five molecules linked to inflammation: in PV, with leptin (inverse correlation) and macrophage-produced IL-1β, IP-10 and IL-1Rα (positive correlations) and in ET, with IL-33 (positive correlation). Since leptin requires JAK2 for its signaling, a negative feed-back is likely to explain the inverse correlation between leptin and the %*JAK2*V617F in PV. No link was found between the %*JAK2*V617F and 37/42 cytokines and receptors.

### 2.6. Inflammation-Linked Cytokines Associated with CALR Exon 9 Mutated-ET

Fifteen ET patients with *CALR* mutation were examined; the characteristics of *CALR*-mutated vs. *JAK2*V617F-mutated ET patients are shown in Supplementary Table S2. As published, *JAK2*V617F-mutated ET patients had a significantly higher hematocrit, and *CALR*-mutated ET patients had higher platelet counts [47].

**Figure 3:**
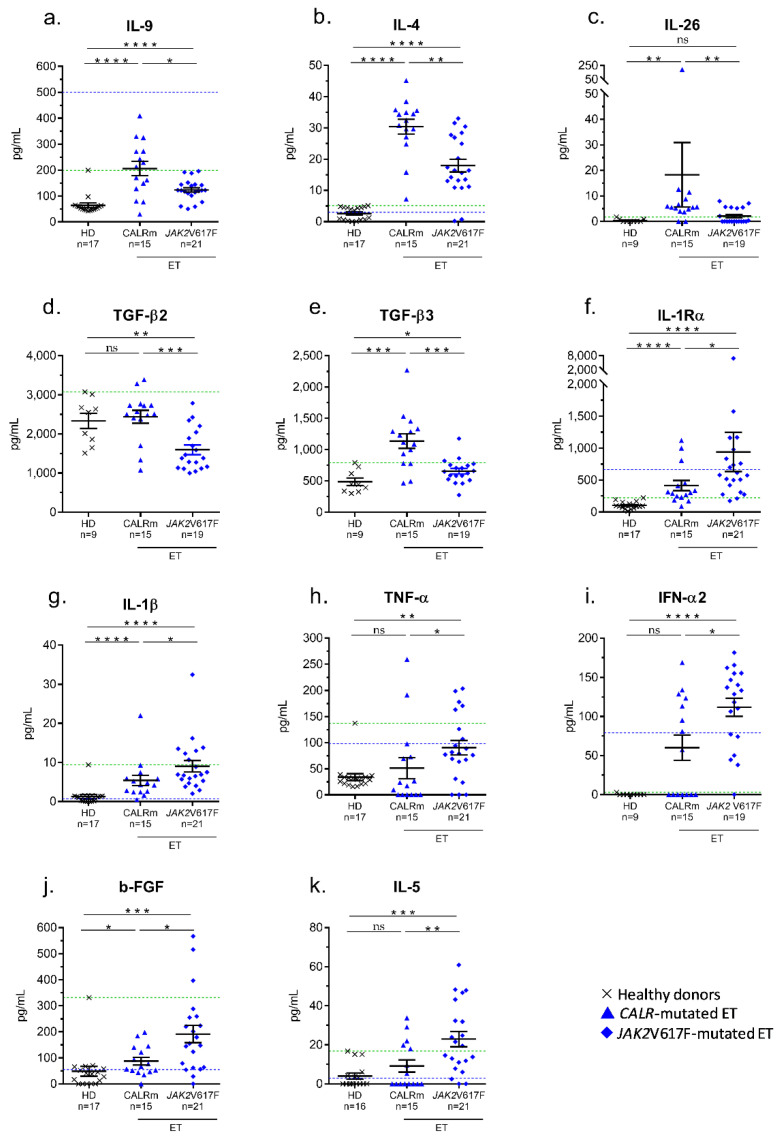
Differences in cytokine levels in *CALR*- and *JAK2*V617F-mutated ET. Significant differences were found between *CALR*- and *JAK*2V617F- mutated ET for 9 cytokines: (**a**) IL-9; (**b**) IL-4; (**c**) IL-26; (**d**) TGF-β_2_; (**e**) TGF-β_3_; (**f**) IL-1Rα; (**g**) IL-1β; (**h**) TNF-α; (**i**) IFN-α_2_; (**j**) b-FGF; (**k**) IL-5. Results are presented as the means + SEM; note the changes in scales (Y axis). (*) *p* < 0.05, (**) *p* < 0.01, (***) *p* < 0.001 and (****) *p* < 0.0001, Mann–Whitney *t*-test. Dotted blue lines represent the upper normal values for healthy individuals according to the manufacturers of the BioPlex Pro-human Cytokine kits, measured in 66 healthy donors. Dotted green lines represent the upper normal values for healthy individuals as measured in our control cohort of 17 healthy donors.

The serum levels of cytokines of ET cohorts were compared. *CALR*-mutated ET patients showed significantly higher levels of three molecules produced by T-cell subsets (IL-4, IL-9, IL-26), plus TGF-β_2_ and TGF-β_3_ (Figure 3a–e). Compared to *JAK2*V617F-mutated ET, *CALR*-mutated ET patients also tended to have high levels of TGF-β_1_ and IL-33, but differences were not significant (*p* = 0.070 and *p* = 0.065, respectively; Mann–Whitney *t*-test). As observed in PV, *JAK2*V617F-mutated ET was characterized by high levels of IL-1Rα and macrophage-derived IL-1β and TNF-α (Figure 3f–h). Patients with *JAK2*V617F-mutated ET also had high levels of IFN-α_2_, b-FGF and IL-5 (Figure 3i–k).

### 2.7. Production of Cytokines by UT-7 Cells Expressing the JAK2V617F or CALR Mutants

We then investigated whether the expression of *JAK2*V617F, tested at different levels in vitro, could affect cytokine production. For these studies, we used a cytokine-dependent human cell line with wild-type JAK2 and CALR. The human UT-7 cell line was chosen because it was obtained from an ET patient, and ET megakaryocytes are a major source of inflammatory cytokines. The UT-7 cell line was preferred to erythroid cell lines because of its capacity to produce and secrete cytokines of interest in MPNs.

**Figure 4:**
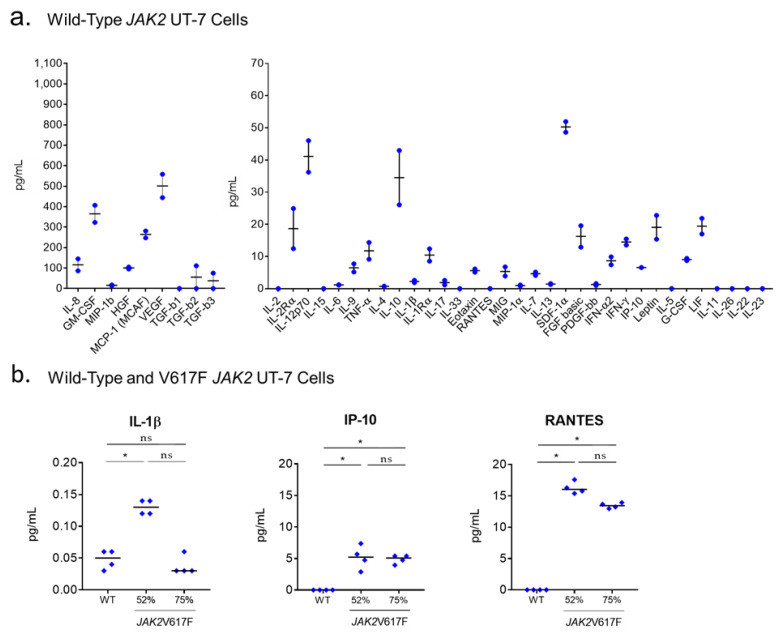
Cytokine production of wild-type *JAK2* and *JAK2*V617F UT-7 cells. (**a**) The basal level of 40 cytokines and chemokines and two soluble receptors were quantified in the supernatants of wild-type *JAK2* UT-7 cells; note the difference in scale for nine cytokines, produced at high levels (left panel). Results are presented as the means + SEM. (**b**) Only three molecules out of 42 measured in quadriplates in cell supernatants were found to be significantly more secreted in the supernatants of UT-7 clones expressing at least 50% *JAK*2V617F, compared to wild-type *JAK2* UT-7 cells; note the difference in scale for IL-1β, produced at very low levels by UT-7 cells (left panel). Median values are represented by black bars. (*) *p* < 0.05, Mann–Whitney *t*-test. In these experiments, UT-7 cells were grown in the presence of GM-CSF.

Firstly, we determined that wild-type UT-7 cells secrete 27/42 cytokines or chemokines, as measured in cell supernatants, including 5/8 cytokines overexpressed in ET compared to PV (IL-9, IL-17, MIG, TGF-β_2_, TGF-β_3_) (Figure 4a). Secondly, the CRISPR/Cas-9 technology was used successfully to obtain UT-7 cells with *JAK2*V617F-mutated alleles (see Methods). Since UT-7 cells have four copies of the *JAK2* gene, introduction of the *JAK2*V617F mutation was expected to generate UT-7 clones with 25%, 50%, 75% or 100% *JAK2*V617F-mutated alleles, as assessed by quantitative allele-specific PCR in gDNA [44,48,49]. However, we only obtained clones with 50% or 75% *JAK2*V617F-mutated alleles (Supplementary Figure S4a,b). Signaling studies confirmed activation of the JAK2/STAT5 pathway in *JAK2*V617F-mutated UT-7 clones (Supplementary Figure S4c). When the same 42 molecules linked to inflammation quantified in the blood of patients were measured in the supernatants of *JAK2*V617F-mutated UT-7 clones, increases were noted only for IL-1β, IP-10 and regulated on activation, normal T-cell expressed and secreted (RANTES), compared to wild-type *JAK2* UT-7 cell supernatants (Figure 4b).

Thirdly, we investigated the effect of *CALR* exon 9 mutants on cytokine production in UT-7 cells, here using UT-7 cell lines that were previously obtained by stable transfection of two plasmid constructs, one containing a cDNA encoding MPL (because CALR requires MPL to exert its action), the other encoding either type 1 (del52) or type 2 (ins5) *CALR* exon 9 mutants [50]. These UT-7 cell lines were grown in the presence of TPO. Expression of *CALR* exon 9 mutants was weak in UT-7 cells yet sufficient to activate the JAK2/STAT5 pathway [50]. Expression of *CALR* mutants in UT-7 cells did not increase the levels of cytokines or receptors secreted in cells supernatants (Supplementary Figure S5). In particular, in contrast to previous reports, in UT-7 cell expression of *CALR* mutants did not alter IL-6 and TNF-α levels [51]. To the opposite, reduced levels of several cytokines (IL-8, MIP-1α, MIP-1β, IP-10) were noted in UT-7 cells expressing type 1 or type 2 *CALR* exon 9 mutants.

To summarize, in MPN patients and in UT-7 cells, no link was found between the production of 37/42 cytokines and *JAK2*V617F expression. Five molecules were either induced by *JAK2*V617F in UT-7 cells (IL-1β, IP-10, RANTES) and/or correlated with the %*JAK2*V617F (IL-1Rα, IL-1β, IP-10, IL-33) in *JAK2*V617F-mutated MPNs. In *JAK2*V617F-mutated MPNs, mutated monocytes–macrophages presumably constitute the main source of IL-1β and IP-10, and also express IL-1Rα. In contrast, three cytokines (IL-4, IL-9, IL-26) produced by T-cell subsets—typically not mutated in MPNs—were associated with *CALR*-mutated ET in patients. Logically, *CALR* mutants did not induce IL-4, IL-9 or IL-26 in UT-7 cells. Our study also confirmed the presence of high levels of HGF and IL-15 in PMF [52,53]. HGF is a survival factor produced autocrinely by most cells, including MPN progenitors, and a marker of short survival in PMF [52,53,54].

### 2.8. Auto-Immunity Against Glucosylsphingosine (GlcSph) in MPN Patients

Chronic inflammation of various origins, including auto-immune disease, is associated with an increased risk of developing an MPN [30,31,32,33]. Intriguingly, glucosyl-sphingosine (GlcSph), a proinflammatory lysosphingolipid accumulated in Gaucher disease (GD), is the target of auto-antibodies in MGUS and in myeloma [34,35,36,37,38]. GD patients also present with chronic inflammation, with high levels of IL-1β, HGF, IL-8, MIP-1β and TNF-α, and an increased risk of malignancy [39,40,41,42,43]. We investigated whether MPN patients may also present with auto-antibodies directed at GlcSph.

#### 2.8.1. Detection of GlcSph-Reactive IgGs in the Serum of MPN Patients

**Figure 5:**
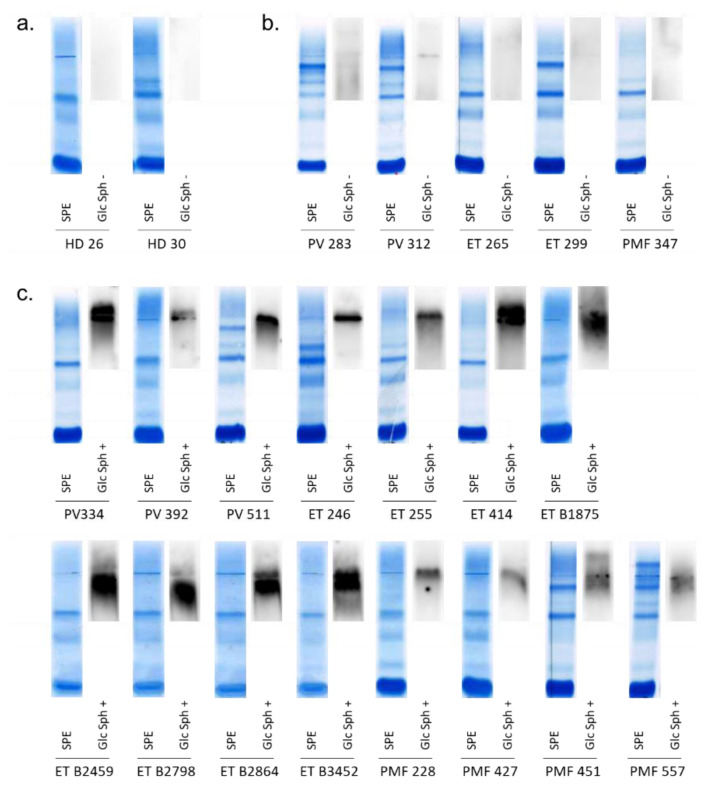
GlcSph-reactivity of serum IgGs from PV, ET, PMF patients. GlcSph-specific immunoblotting assays were performed as described in Methods. (a,b,d) Both the gel of serum protein electrophoresis (SPE), after coloration, and the result of the GlcSph immunoblot, revealed by chemiluminescence, are shown; note that migration pattern may vary. (**a**) Example of 2/54 HDs with no GlcSph-reactive IgG in serum. (**b**) Examples of 5/60 MPN patients with no GlcSph-reactive IgG in serum. (**c**) 15/15 MPN patients with GlcSph-reactive IgGs in serum.

Serum samples of the 75 MPN patients and 54 HDs were used to analyze the eventual GlcSph-specificity of serum IgGs, using a home-made GlcSph (LGL1) immunoblot assay (see Methods) adapted from Nair et al. [34,35,36,37,38,55,56]. As shown in Figure 5, the presence of GlcSph-reactive IgGs in the serum was observed for 15/75 (20%) MPN patients, and for only 2/54 (3.7%) HDs (*p* = 0.0073 Fisher exact test).

The results of the GlcSph assays were negative for 60 MPN patients and positive for 15 MPN patients (Figure 5). Among the 15 patients with GlcSph-reactive IgGs, there were 3/27 PV (11.1%), 8/39 ET (20.5%) and 4/9 PMF (44.4%). Thus, in this cohort, the presence of GlcSph-reactive IgGs was more frequent in PMF than in PV (*p* = 0.0497, Fisher exact test); the difference in frequency between PMF and ET was not significant. Among those 15 patients, 9 carried the *JAK2*V617F mutation (3 PV, 3 ET, 3 PMF), 5 had a *CALR* mutation (4 ET, 1 PMF) and 1 was an *MPL*-mutated ET. The frequencies of GlcSph-reactive IgGs in *CALR*-mutated MPNs was 31.3% (5/16) vs. 16.4% (9/55) for *JAK2*V617F-mutated MPNs, but the difference was not significant (*p* = 0.2818, Fisher exact test).

#### 2.8.2. Characteristics of MPN Patients with GlcSph-Reactive IgGs

Possibly due to the small size of the cohort of patients with GlcSph-reactive IgGs, we found no significant difference in age or blood parameters between MPN patients with and without GlcSph-reactive IgGs (Supplementary Table S3). Overall, the %*JAK2*V617F of *JAK2*V617F-mutated patients with GlcSph-reactive IgGs was low—always ≤37% except for one PV with 96% *JAK2*V617F.

**Figure 6:**
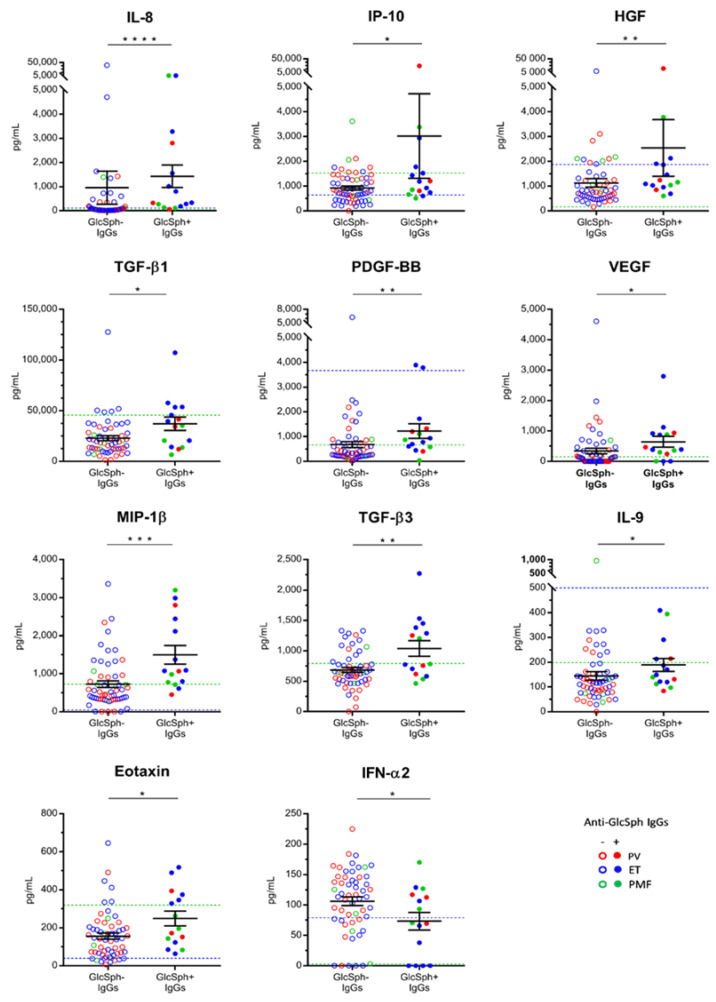
Cytokine levels according to the presence of GlcSph-reactive IgGs. Significant differences in the serum levels of 11 cytokines were found between MPN patients with GlcSph-reactive IgGs (+) and MPN patients with no GlcSph-reactive IgGs (-), as indicated. Results are presented as the means + SEM. (*) *p* < 0.05, (**) *p* < 0.01, (***) *p* < 0.001 and (****) *p* < 0.0001, Mann–Whitney *t*-test. Dotted blue lines represent the upper normal values observed for healthy individuals according to the manufacturers of the BioPlex Pro-human Cytokine kits, measured in 66 healthy donors. Dotted green lines represent the upper normal values for healthy individuals as measured in our control cohort of 17 healthy donors.

We then analyzed the cytokine levels of patients: compared to other MPN patients, the 15 patients with GlcSph-reactive IgGs had increased levels of 10 cytokines: IL-8, IP-10, HGF, TGF-β_1_, TGF-β_3_, platelet-derived growth factor-BB (PDGF-BB), VEGF, macrophage inflammatory protein 1β (MIP-1β), IL-9 and eotaxin (Figure 6). Interestingly, perhaps because of a negative feed-back mechanism in an auto-immune context, MPN patients with GlcSph-reactive auto-antibodies had a reduced level of IFN-α_2_, an immunostimulant (Figure 6).

#### 2.8.3. Analysis of GlcSph Levels in the Serum of MPN Patients

**Figure 7:**
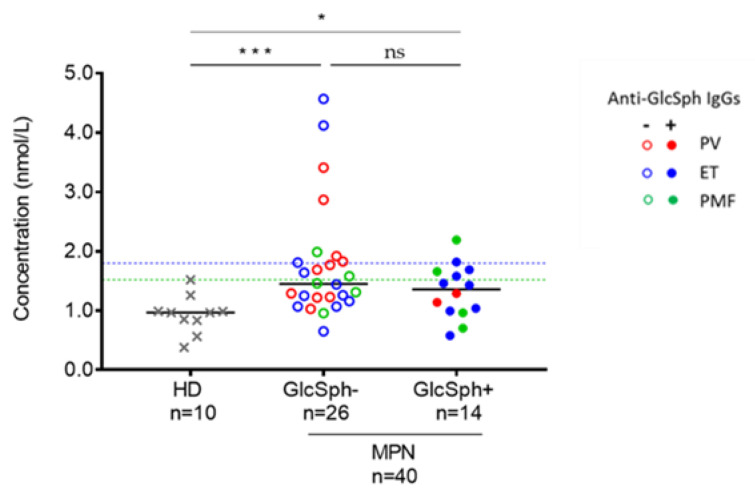
GlcSph levels measured in the blood serum of MPN patients. GlcSph−: MPN patients without GlcSph-reactive IgGs; GlcSph+: MPN patients with GlcSph-reactive IgGs. (*) *p* < 0.05, (***) *p* < 0.001, Mann–Whitney *t*-test. Median values are represented by black bars. The dotted blue line represents the upper normal value for healthy individuals (1.8 nmol/L). The dotted green line represents the upper value of our control cohort of HDs.

GlcSph-reactive autoantibodies were first described in the context of GD, characterized by the accumulation of glucocerebroside and GlcSph [40]. We used a previously published technique [57] to measure the level of GlcSph in the blood serum of 40 MPN patients (12 PV, 19 ET, 9 PMF). Among these patients, 14 had GlcSph-reactive IgGs and 26 did not. GlcSph values of healthy adults are always <1.8 nmol/L [57]. Accordingly, GlcSph levels in our HD series were all ≤1.5 nmol/L (Figure 7). For MPN patients, the median GlcSph value was 1.4 nmol/L (range: 0.6–4.6 nmol/L). Seventeen MPN patients (42.5%) had a GlcSph level >1.5 nmol/L (6 PV, 7 ET, 4 PMF). A GlcSph level >1.8 nmol/L was observed for 10 (25%) MPN patients (4 PV, 4 ET, 2 PMF) (Figure 7).

We analyzed GlcSph levels according to the presence of GlcSph-reactive IgGs. There was no significant difference between patients with GlcSph-reactive IgGs (median GlcSph level: 1.35 nmol/L, range: 0.6–2.2) and those without (median GlcSph level: 1.45 nmol/L, range: 0.7–4.6), but both groups of MPN patients had significantly higher GlcSph levels than healthy controls (Figure 7). We noted a tendency to less increased GlcSph levels in patients with anti-GlcSph antibodies: only 1/14 (7.1%) MPN patients with anti-GlcSph IgGs had a GlcSph level > 1.8 nmol/L vs. 8/26 (30.8%) MPN patients without anti-GlcSph IgGs (difference not significant, *p* = 0.1243, Fisher exact test). One may hypothesize that anti-GlcSph autoantibodies contribute to reduce GlcSph levels. We then searched for eventual correlations between the GlcSph level and blood cell counts in MPN patients who carry GlcSph-reactive IgGs, but found no correlation.

## 3. Discussions

**Figure 8:**
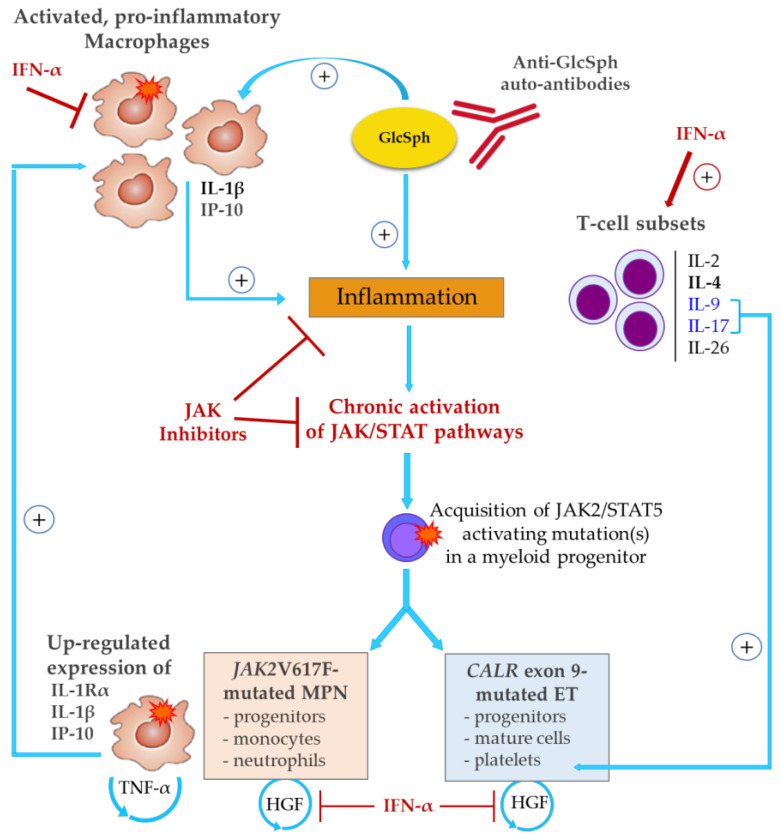
*JAK2*V617F-dependent vs. mutation-independent inflammation in MPNs. At least 26 cytokines are overproduced in MPNs. *CALR* mutants did not alter cytokine production, whereas *JAK*V617F increased the expression of IL-1Rα, IL-1β and IP-10. Other cytokines overexpressed in MPNs are presumably produced by nonmutated cells, and this type of inflammation is likely to precede the acquisition of JAK2/STAT5 activating mutations. An autoimmune response to GlcSph, observed in 20% of MPNs, could be an early cause of chronic inflammation—and excessive IL-1β production—eventually leading to the acquisition of *JAK2* or *CALR* mutations and MPN development. In *JAK2*V617F-mutated MPNs, strongly inflammatory IL-1β associated with IL-1Rα expression, both induced by *JAK2*V617F, further stimulate cytokine production by both nonmutated cells of the bone marrow environment and mutated cells. *JAK2*V617F-mutated cells also secrete TNF-α, known to enhance clonal expansion. Neither IL-1β nor TNF-α signal via JAK/STAT, and thus are not sensitive to JAK inhibitors. In contrast, IFN-α represses IL-1β, and also HGF, an autocrine survival factor for clonal MPN progenitors. In addition, IFN-α stimulates T-lymphocyte subsets that secrete anti-inflammatory IL-4, and two prothrombopoiesis and prothrombosis cytokines—IL-9 (an inducer of IL-4) and IL-17.

Our study provides evidence both in MPN patients and in new human UT-7 cell lines that the impact of the *JAK2*V617F mutation on the production by mutated cells of inflammation cytokines and receptors concerns essentially IL-1β, IL-1Rα and IP-10. Thus, most of the inflammation associated with *JAK2*V617F-mutated MPNs is mutation-independent, i.e., either precedes the acquisition of *JAK2*V617F or is reactive to the mutated clone, or both. Moreover, *CALR* exon 9 mutants had no effect at all on cytokine production in UT-7 cells, and for patients with *CALR*-mutated ET, the main source of the characteristic cytokines found in excess (IL-4, IL-9, IL-26) was nonmutated T-cells. We also report the presence of auto-antibodies directed at pro-inflammatory lysosphingolipid (GlcSph) in 20% of MPNs, particularly in ET and PMF. Altogether, our findings are consistent with the existence of early causes of inflammation in subsets of MPN patients, which result in chronic overstimulation of myelopoiesis via the JAK2/STAT5 pathway and thus, facilitate the acquisition of *JAK2* or *CALR* mutations that further activate JAK2/STAT5 (Figure 8).

Our study confirms that the three subtypes of MPNs present with a very high level of inflammation (26 cytokines produced in excess among 42 measured). The cytokine levels found in the blood of MPN patients were higher than those previously observed in patients with myeloma, although myeloma is a much more severe blood cancer than MPNs [45]. Of note, whereas EPO and TPO are low in MPNs, high levels of JAK2-activating G-CSF (and IL-6) were observed in MPN patients despite the fact that patients typically present with elevated neutrophil counts. The absence of negative feed-back for G-CSF in MPN patients supports the existence of a cytokine-induced (mutation-independent) stimulation of granulomonopoiesis possibly anterior to, and facilitating, the acquisition of mutations (represented in Figure 8).

There are examples of hematological malignancies where inflammation precedes the acquisition of multiple genetic mutations and the subsequent expansion of the mutated clone. Our previous study of inflammation in the context of myeloma and its asymptomatic stage (MGUS) revealed that except for four cytokines found to be significantly higher in myeloma (HGF, IL-11, RANTES, SDF-1α), the levels of 38 inflammation cytokines were similar in MGUS and in myeloma [45]. Thus, a high level of inflammation is already present at the asymptomatic, benign stage of MGUS, and except for four molecules, the acquisition of multiple genetic alterations characteristic of progression toward myeloma did not significantly increase cytokine production. One can envision that in MPNs as well as in myeloma, most of the chronic inflammation could precede mutation and not be solely the consequence of genetic alterations.

Other observations support an early role of cytokines in MPN pathogenesis. Firstly, different MPN subtypes are characterized, independently of their mutation status, by different cytokines: macrophage-produced IL-1β for PV; T-cell-produced IL-4, IL-9, IL-26, TGF-β for ET; IL-15 and anti-inflammatory HGF for PMF. Secondly, neither the *CALR* exon 9 mutant load (typically ~50%) nor the variable *JAK2*V617F mutant load correlate with the blood parameters of patients, when *JAK2*V617F-mutated PV and ET patients are studied separately. In contrast, in PV and in ET, the levels of many cytokines or receptors correlated positively with the blood counts of leukocytes, neutrophils and monocytes (IL-1β, IL-1Rα, HGF, MIG), platelets (IL-9, IL-17, SDF-1α) or lymphocytes (IL-9). Of note, recent reports describe IL-9 as a STAT5 activator that enhances megakaryocytopoiesis, and both IL-9 and IL-17 as prothrombotic cytokines [58,59,60]. In contrast, the PMF cohort was characterized by negative correlations between numerous cytokines and blood counts of leukocytes, neutrophils and monocytes (TNFα, IL-10, IL-15, VEGF, MIP-1β, IL-8, others) and lymphocytes (IL-17, MIP-1α). No correlation was found between cytokine levels and parameters of the red cell lineage in PV and in PMF, but in ET, the hematocrit correlated negatively with the levels of IL-2, IL-4 and IL-26. Thirdly, the cytokine production of human megakaryocytic UT-7 cells was minimally affected by the expression of MPN main mutations: *CALR* exon 9 mutants had no effect at all, and *JAK2*V617F induced only three molecules: IL-1β and IP-10 (both correlated with the %*JAK*2V617F of patients) and RANTES. Interestingly, several teams previously reported that high levels of mostly macrophage-produced IL-1β and IP-10 were predictive markers of a worse prognosis and short survival in MPNs [52,53,61,62]. In addition, like HGF and TNF-α, recent studies suggest that IL-1β favors MPN disease initiation and clonal expansion [54,63,64,65]. In the context of ET, Øbro et al. reported that GRO-α and EGF (not measured in our study) were potential markers of disease progression, independently of *JAK2*V617F or *CALR* mutation [66]. To summarize, the data indicate that IL-1β, IL-1Rα and IP-10 are linked to *JAK2*V617F mutation, whereas all other cytokines in excess in MPNs are produced independently of *JAK2* and *CALR* mutations but differentially according to the MPN phenotype. The blood cell counts of patients correlated with the levels of many cytokines, better than with mutant loads. Hence, the study suggests that for subsets of patients, a prolonged inflammatory response may be the early event eventually leading to an MPN.

There is now clear evidence that certain individuals and families may present with a predisposition for the acquisition of one or more MPN-associated mutations. For MPNs, facilitating backgrounds include tobacco smoking, genetic polymorphisms or alterations, such as specific haplotypes or germline mutations, and auto-immune diseases, all associated with important chronic inflammation [30,31,32,33,67,68,69,70,71]. We report here the first evidence in MPNs of elevated levels of an immunogenic, pro-inflammatory lysosphingolipid, glucosylsphingosine (GlcSph)—also called lyso-glucosylceramide (LGL1), as well as the presence of auto-antibodies that target GlcSph in 20% of MPN patients. The age and blood counts of MPN patients with GlcSph-reactive IgGs did not differ from those of other patients; their %*JAK*2V617F tended to be low. However, MPN patients with GlcSph-reactive IgGs had a distinct cytokine profile, with significantly higher levels of IL-8, IP-10, HGF, TGF-β_1_, TGF-β_3_, PDGF-BB, VEGF, MIP-1β, IL-9 and eotaxin, and low levels of IFN-α_2_.

Analysis of the GlcSph levels in the blood serum of 40 MPN patients revealed moderately elevated GlcSph levels in all three subtypes in MPNs, but there was no correlation of the GlcSph level with the presence of anti-GlcSph autoantibodies. Accumulation of GlcSph is a key biomarker of GD, and the consequence of germline mutations in the glucocerebrosidase (GBA) gene. GD patients affected with the most common visceral form of the disease have splenomegaly, hepatomegaly, bone lesions, cytopenia and chronic inflammation with elevated levels of IL-1, IL-6, IL-8 and TNF-α [72]. However, patients may also be asymptomatic, and atypical GD may be diagnosed only late in life. GD patients present with an increased risk of cancer, including hematological malignancy, notably B-cell lymphoma, myeloma and also myeloid neoplasms [40,41,42,43]. Recent studies have established that GlcSph is a frequent target of both clonal and nonclonal immunoglobulins of GD patients who develop a MGUS or a myeloma, which implies that auto-immune responses to GlcSph do initiate cases of MGUS and myeloma [34,35,36,37,38]. In strong support of a GlcSph-driven MGUS/myeloma pathogenic process, Nair et al. showed that for two patients, GlcSph reduction therapy (by Eliglustat) successfully suppressed the monoclonal immunoglobulin [36]. In the present retrospective study, we were not able to analyze patient DNA for germline mutations in the GBA gene. Further studies are thus required to determine whether MPN patients with abnormal levels of GlcSPh and/or anti-GlcSph autoantibodies carry alterations in the GBA gene.

In conclusion, most of the inflammation-linked cytokines found in excess in MPNs were not linked to *JAK2*V617F nor to *CALR* mutation; only IL-1β, IL-1Rα and IP-10 were induced by *JAK2*V617F. Precisely via the action of circulating IL-1β and IP-10 in the blood or bone marrow micro-environment, it is possible that *JAK2*V617F indirectly affects the production of other inflammatory cytokines, particularly by macrophages since these cells are a major source of IL-1β, IP-10 and to a lesser degree, of RANTES. These observations have important consequences for therapy, since they imply that in addition to JAK inhibitors, blocking the most important cytokines in MPNs (IL-1β, TNF-α, HGF, all insensitive to JAK/STAT inhibitors) may be considered [65,73,74]. IFN-α therapy achieves this in part via the repression of IL-1β and HGF. However, other causes of inflammation eventually leading to an MPN should be actively searched. One may have auto-immunity against GlcSph. Since GlcSph can be reduced with existing treatments, this lysosphingolipid could also become a useful new target in MPN therapy for selected patients.

## 4. Materials and Methods

### 4.1. Patients

The study was performed with the approval of local ethical committees and the Commission Nationale de l’Informatique et des Libertés (CNIL #912335). Written informed consents were obtained from patients in the relevant clinical departments, and in the blood bank for healthy volunteers enrolled by the Établissement Français du Sang (EFS, Nantes, France). An agreement was signed between our laboratory (CRCINA, Inserm U1232) and the blood bank (EFS Pays de La Loire). Seventy-five patients presenting with an MPN (27 PV, 39 ET, 9 PMF), diagnosed at the French University hospitals of Nantes and Brest (BB-0033-00037, CRB Santé du CHRU de Brest), according to the World Health Organization (WHO) 2016 criteria, were included in this study. Sera from 54 healthy volunteers, obtained from EFS Pays de la Loire, were also studied as controls. The mutational status of patients was established in hospital laboratories. Serum samples were aliquoted and kept frozen at −80 °C until use. Due to occasional insufficient sample collection, all assays were not performed for all patients.

### 4.2. UT-7 Cells

Human megakaryocytic UT-7 cells were grown at 37 °C with 5% CO_2_ in Minimum Essential Media (MEM) or in Iscove’s Modified Dulbecco’s Media (IMDM) and 10% foetal calf serum (FCS), in the presence of either GM-CSF (5 ng/mL) or TPO (10 ng/mL) (PeproTech France, Neuilly sur Seine, France). UT-7 cell lines expressing the type 1 (del52) or type 2 (ins5) *CALR* exon 9 mutants were grown in the presence of TPO and doxycyline (Sigma) to induce the expression of the transgene, as published [50]. For cytokine studies, UT-7 cells were washed twice, plated at 10^5^/mL in 6-well plates in duplicates or quadriplates, and incubated for 18 h at 37 °C with 5% CO_2_. Cells were then centrifuged, supernatants were collected, aliquoted and kept frozen at −80 °C.

### 4.3. Generation of JAK2V617F+ UT-7 Cells Using the CRISPR/CAS-9 Technology

Gene editing to introduce the sequence encoding of the *JAK2V617F* mutation in UT-7 cells was performed using a CRISPR-Cas9 based approach. A high-scoring single guide RNA (sgRNA) targeting exon 14 of the human *JAK2* gene was designed using the CRISPOR portal (http://crispor.tefor.net/). A sgRNA with optimal predicted cutting efficiency and safety profile, generating a double strand break only 1 bp away from the targeted insertion site for optimal mutagenesis, was selected (20 nucleotide targeting sequence + PAM = AATTATGGAGTATGTGTCTG TGG). The sgRNA, ordered from Synthego as a chemically modified synthetic RNA molecule, was then complexed with purified wildtype Sp. Cas9 protein (New England Biolabs) at a 3:1 sgRNA: Cas9 molar ratio to form a ribonucleoprotein complex (RNP). UT-7 cells were then electroporated using a Lonza nucleofector device following the manufacturer’s recommendations with 2.5 µM RNP and 1µM 127-mer ssODN encoding the *JAK2V617F* mutation flanked by asymmetric homology arms as a repair template (TCCTGA AACTGAATTTTCTATATAAACAAAAACAGATGCTCTGAGAAAGGCATTAGAAAGCCTGTAGTTTTACTTACTCTCGTCTCCACAGAAACATACTCCATAATTTAAAACCAAATGCTTGTGA). Postelectroporation, modified UT-7 cells were plated in 96-well plates using a limiting dilution technique, and subclones were tested for the presence of the *JAK2V617F* mutation using deconvolution of Sanger sequencing trace profiles (TIDE algorithm) on PCR amplicons generated from crude genomic DNA extracts (FwdSeqhJak2Ex14: CAGTTGCAGGTCCATATAAAGGGACC and RvseSeqhJak2Ex14: CCAGTTATTCCAATGTTATGTTGAACCTGCC); then, the presence and quantity of *JAK2*V617F was assessed using *JAK2*V617F allele-specific quantitative PCRs [44,48,49]. UT-7 clones positive for the *JAK2V617F* mutation were then minimally expanded and cryo-preserved until further analysis.

### 4.4. Quantification of JAK2V617F in UT-7 Clones

Genomic DNA was prepared using a QiaAmp DNA mini-kit (Qiagen, Valencia, CA) and *JAK2* wild type (WT), and *JAK2*V617F allele-specific quantitative PCRs (AS-qPCRs) were performed in genomic DNA as published [44,48,49].

### 4.5. Quantification of Inflammation-Linked Cytokines

Frozen aliquots of serum from MPN patients and HDs and UT-7 cell supernatants, were used to quantify 40 chemokines and cytokines and 2 soluble cytokine receptors linked to inflammation using the Luminex technology (Bio-Plex 200) with Bio-Plex Pro Human Cytokine Panel kits (Bio-Rad, Hercules, CA, USA), as published [45].

### 4.6. Detection of GlcSph-Reactive Igs

Analysis of the presence of IgGs specific for GlcSph (LGL1) was performed using an immunoblotting assay adapted from Nair et al. [34,35,36,37,38]. Polyvinylidene fluoride (PVDF) membranes were incubated for 90 min in 100μg/mL of GlcSph in 0.1 M sodium bicarbonate, rinsed 3 times in phosphate buffer saline (PBS) and 0.1% Tween 20 detergent, then blocked for 2 h with 5% bovine serum albumin (BSA) in PBS and 0.1% Tween 20. Samples of serum were submitted to agarose gel electrophoresis; then, the gels were blotted onto the GlcSph-saturated membranes by diffusion blotting during 12 min [55,56]. After blocking for 1 h with 2.5% BSA in PBS and 0.1% Tween 20, membranes were incubated with peroxidase-conjugated AffiniPure donkey antihuman IgG (H + L) antibody (Jackson ImmunoResearch, West Grove, PA, USA) or horseradish peroxidase (HRP)-conjugated goat anti-human IgA α chain antibody (Bethyl Laboratories, Montgomery, TX, USA) for 1 h, then washed and revealed with Super Signal West Pico chemiluminescent substrate (Thermo Scientific, Waltham, MA, USA).

### 4.7. GlcSph Quantification

Patient serum (200 µL) was mixed rapidly with 500 µL phosphoric acid and 500 µL methanol containing 50 nM internal standards. Supernatants were purified by solid phase extraction (MCX Oasis cartridge). Eluates were evaporated under N2 and reconstituted with the mobile phase. An amount of 25 µL was injected in LC-MS/MS (API 4500 QTrap, ABsciex, Foster, CA, USA). The quantification (nmol/L) was performed by Analyst software using external standard calibration [57].

### 4.8. Statistics

Data analysis was performed by GraphPad Prism 7.05 software. Patient parameters were expressed as the medians and ranges, and/or the means ± standard error of the mean (SEM). The Chi-2 test was used for categorical variables. For continuous variables (*n* < 30), a normality test was systematically performed for each group. For nonparametric conditions, a Mann–Whitney *t*-test was performed. The tests used are indicated in the legends of Figures and Tables. A *p* value below 0.05 was considered statistically significant.

## Figures and Tables

**Table 1:** Characteristics of myeloproliferative neoplasm (MPN) Patients.

	All Patients	PV	ET	PMF
**Number**	75	27	39	9
**Sex**, M/F (male%)	38/37 (50.7%)	16/11 (59.3%)	15/24 (41.0%)	7/2 (77.8%)
**Age** (Yr)				
Median	67.0	69.0	73.0	62.0
(Range)	(33–95)	(33–93)	(37–95)	(50–86)
%***JAK2*V617F**				
Median	16.0	42.0	5.0	27.0
(Range)	(0–96)	(7–96)	(0–61 *)	(0–51)
**Blood Counts**				
Hematocrit (L/L)				
Median	46.3	53.4	44.1	36.2
(Range)	(25.1–70.0)	(43.1 **–70.0)	(35.3–63.3 ***)	(25.1–41.0)
Hemoglobin (g/dL)				
Median	14.8	17.4	14.4	11.0
(Range)	(7.9–22.4)	(13.8 **–22.4)	(10.4–20.0 ***)	(7.9–13.0)
Leukocytes (×10^9^/L)				
Median	8.7	9.7	8.3	11.3
(Range)	(2.8–41.0)	(2.8–37.8)	(4.5–41.0)	(5.0–27.5)
Platelets (×10^9^/L)				
Median	527.5	377	644	165
(Range)	(47–2300)	(89–851)	(191–2300)	(47–586)

* Thrombocythemia (ET) patient with post-ET myelofibrosis; ** polycythemia vera (PV) patient who had received treatment at the time of serum cytokine studies; *** ET which transformed into PV.

**Table 2:** Cytokine levels in MPN patients.

Molecules (pg/mL)	HD n = 17		All MPNs n = 75		PV n = 27		ET n = 39		PMF n = 9	
	Med.	(Range)	Med.	(Range)	Med.	Range	Med.	(Range)	Med.	(Range)
Markers of poor prognosis in MPNs										
**sIL-2Rα**	69.8	(0.0–162)	**295.9**	(30.3–1438)	287.2	(39.5–1129)	275.6	(30.3–1438)	382.6	(205–601)
**IL-8**	15.4	(3.0–43.1)	**80.2**	(2.4–40,808)	54.1	(2.4–2804)	85.5	(14.4–40,808)	120.4	(13.8–5093)
IL-12p70	13.4	(1.4–277)	78.0	(0.0–789)	76.3	(0.0–378)	86.2	(0.0–789)	81.8	(0.0–303)
**IL-15**	0.0	(0.0–0.0)	**0.0**	(0.0–94.4)	0.0	(0.0–55.7)	0.0	(0.0–90.9)	0.0	(0.0–94.4)
GM-CSF	0.0	(0.0–2.11)	0.0	(0.0–382)	0.0	(0.0–156)	0.0	(0.0–266)	0.0	(0.0–382)
**MIP-1β**	310.3	(1.34–727)	**612.7**	(0.0–3362)	448.1	(0.0–2802)	701.6	(1.7–3362)	714.2	(292–3199)
Anti-inflammation										
**IL-4**	3.6	(0.0–5.1)	**20.2**	(0.0–45.1)	13.8	(0.0–35.8)	26.9	(0.2–45.1)	21.3	(8.1–40.7)
IL-10	0.0	(0.0–166)	15.8	(0.0–152)	24.8	(0.0–103)	1.5	(0.0–152)	22.6	(0.0–86.3)
**HGF**	302.6	(125–471)	**837.4**	(166–18,303)	815.0	(165–18,303)	815.6	(286–9302)	1158	(600–3778)
Pro-inflammation										
**TNF-α**	29.7	(25.7–38.7)	**78.9**	(0.0–294)	88.4	(0.0–294)	69.2	(0.0–259)	105.0	(0.0–191)
**IL-1β**	1.0	(0.0–9.4)	**6.2**	(0.5–42.5)	7.4	(1.0–25.8)	5.8	(0.5–42.5)	4.8	(1.3–19.2)
**IL-1Rα**	96.9	(13.5–222)	**605.0**	(87.6–6884)	966.3	(105.2–5284)	500.8	(87.6–6884)	480.3	(193.2–2570)
**IL-2**	0.0	(0.0–3.42)	**0.0**	(0.0–152)	0.0	(0.0–59.9)	0.0	(0.0–115)	0.0	(0.0–152)
**IL-6**	0.8	(0.0–7.6)	**16.9**	(0.0–623)	16.7	(0.0–49.9)	15.9	(0.0–623)	25.8	(10.4–52.8)
**IL-7**	0.0	(0.0–10.6)	**31.9**	(0.0–190)	30.2	(0.0–89.7)	33.7	(0.0–190)	33.9	(5.0–69.7)
**IL-9**	53.2	(42.6–198.7)	**122.0**	(0.0–962)	96.9	(0.0–289)	141.6	(29.0–409)	112.0	(38.5–962)
IL-11	0.0	(0.0–0.0)	0.0	(0.0–0.1)	0.0	(0.0–0.1)	0.0	(0.0–0.0)	0.0	(0.0–0.0)
IL-13	1.2	(0.0–6.49)	5.7	(0.0–83.6)	16.1	(0.0–42.3)	1.1	(0.0–65.0)	0.0	(0.0–83.6)
**IL-17**	124.1	(0.0–255)	**575.0**	(0.0–1240)	529.0	(0.0–968)	648.2	(0.0–1240)	371.3	(0.0–1089)
IL-22	0.0	(0.0–0.0)	0.0	(0.0–0.0)	0.0	(0.0–0.0)	0.0	(0.0–0.0)	0.0	(0.0–0.0)
IL-23	0.0	(0.0–2.8)	0.0	(0.0–6.2)	0.0	(0.0–0.0)	0.0	(0.0–6.2)	0.0	(0.0–0.0)
IL-26	0.0	(0.0–1.71)	3.2	(0.0–195)	0.0	(0.0–7.1)	4.9	(0.0–195)	5.2	(0.0–46.4)
**IL-33**	0.27	(0.0–3.46)	**0.0**	(0.0–11.1)	0.0	(0.0–11.1)	0.0	(0.0–5.8)	0.0	(0.0–7.6)
**IFN-α2**	0.0	(0.0–2.9)	**110.5**	(0.0–225)	115.9	(0.0–225)	106.6	(0.0–182)	93.3	(3.1–170)
**IFN-γ**	17.2	(2.0–34.7)	**143.8**	(0.0–689)	156.5	(0.0–690)	147.0	(0.0–509)	129.3	(0.0–498)
IP-10	626.2	(298–1528)	847.6	(0.0–26,760)	1161	(0.0–26,760)	721.4	(189–2935)	847.6	(442–3613)
Eotaxin	111.5	(44.2–319)	146.5	(6.1–646)	141.7	(6.1–491)	169.3	(19.4–646)	143.4	(28.0–2623)
**FGF basic**	38.5	(0.0–332)	**121.2**	(0.0–567)	128.6	(0.0–405)	121.2	(0.0–567)	111.3	(0.0–332)
Leptin	2206	(584–10,200)	2713	(463–36,254)	2449	(552–22,567)	2.84	(2840–2816)	1781	(733–4489)
**MCP-1**	0.0	(0.0–123)	**0.0**	(0.0–1524)	0.0	(0.0–197)	0.0	(0.0–1524)	0.0	(0.0–1211)
**MIG**	325.3	(211–1193)	**699.7**	(0.0–5878)	477.3	(0.0–2820)	777.8	(312–5605)	1059	(516–5878)
MIP-1α	5.0	(1.0–615)	13.4	(0.0–848)	13.7	(0.0–848)	13.2	(0.0–620)	10.1	(0.0–18.2)
PDGF-BB	464	(34.1–666)	418.2	(25.8–6263)	350.3	(25.8–2181)	603.7	(41.4–6263)	232.1	(34.9–1107)
RANTES	13,592	(3108–25,751)	12,342	(724–66,241)	11,132	(724–48,946)	14,943	(3372–66,241)	18,805	(2451–33,031)
**SDF-1α**	750	(610–970)	**1093**	(0.0–1769)	1093	(0.0–1602)	1106	(540–1769)	1,03	(360–1416)
**VEGF**	0.0	(0.0–144)	**147.6**	(0.0–4605)	124.3	(0.0–1443)	174.7	(0.0–4605)	280.0	(0.0–880)
Other hematopoietic growth factors										
**IL-5**	0.0	(0.0–16.8)	**13.8**	(0.0–84.6)	17.8	(0.0–84.6)	13.0	(0.0–60.9)	18.6	(0.0–49.3)
**G-CSF**	12.2	(0.0–16.8)	**123.1**	(0.0–265)	123.1	(0.0–265)	122.8	(0.0–212)	156.6	(38.7–218)
LIF	0.0	(0.0–4.52)	41.5	(0.0–299)	56.1	(0.0–292)	27.6	(0.0–299)	6.4	(0.0–275)
TGF-β1	16,765	(4928–47,560)	20,530	(1355–127,571)	13,639	(1355–41,498)	27,802	(7782–127,571)	17,741	(6566–35,311)
**TGF-β2**	2554	(1512–3078)	**1647**	(439–3386)	1195	(439–2983)	2203	(996–3386)	1351	(1154–2721)
**TGF-β3**	441.6	(300–790)	**659.6**	(0.0–2268)	577.2	(0.0–1260)	772.7	(275–2268)	638.2	(462–1201)

The median serum levels of 26 cytokines (indicated in blue) were significantly higher for MPN patients (“all MPNs”) than for healthy donors (*p* < 0.05, Mann–Whitney *t*-test).

**Table 3:** Correlations between cytokine levels and blood counts in PV, ET and PMF.

Molecules	Dg	Neutrophils			Monocytes			Hematocrit			Platelets			Lymphocytes		
		n	r	*p* Value	n	r	*p* Value	n	r	*p* Value	n	r	*p* Value	n	r	*p* Value
**IL-1Rα**	PV	24	0.800	*<0.0001*		-	*-*		-	*-*		-	*-*		-	*-*
IL-1β	PV	24	0.496	*0.0137*		-	*-*		-	*-*		-	*-*		-	*-*
IP-10	PV	24	0.476	*0.0186*	25	0.576	*0.0026*		-	*-*		-	*-*		-	*-*
**IL-8**	PMF		-	*-*	9	−0.786	*0.0279*		-	*-*		-	*-*		-	*-*
**MIP-1α**	PMF		-	*-*		-	*-*		-	*-*		-	*-*	9	−0.810	*0.0218*
**MIP-1β**	PV		-	*-*		-	*-*		-	*-*		-	*-*	24	0.440	*0.0314*
	PMF		-	*-*	9	−0.738	*0.0458*		-	*-*		-	*-*	9	−0.762	*0.0368*
**Leptin**	PV	15 M	−0.600	*0.0223*		-	*-*		-	*-*		-	*-*		-	*-*
	PMF		-	*-*	8	−0.786	*0.0279*		-	*-*		-	*-*		-	*-*
**IL-2Rα**	ET		-	*-*		-	*-*		-	*-*	38	0.613	*0.0052*		-	*-*
**SDF-1α**	PV		-	*-*		-	*-*		-	*-*	24	0.605	*0.0017*		-	*-*
	ET		-	*-*		-	*-*		-	*-*	38	0.520	*0.0012*		-	*-*
IL-2	ET		-	*-*		-	*-*	38	−0.519	*0.0009*		-	*-*		-	*-*
IL-4	ET		-	*-*		-	*-*	38	−0.464	*0.0034*		-	*-*	38	0.439	*0.0065*
IL-7	PV		-	*-*		-	*-*		-	*-*	24	0.525	*0.0070*		-	*-*
IL-9	PV		-	*-*		-	*-*		-	*-*		-	*-*	24	0.412	*0.0454*
	ET		-	*-*		-	*-*		-	*-*	38	0.420	*0.0087*	38	0.451	*0.0050*
IL-17	PV		-	*-*		-	*-*		-	*-*	24	0.508	*0.0095*		-	*-*
	ET		-	*-*		-	*-*		-	*-*	38	0.468	*0.0030*		-	*-*
IL-26	ET		-	*-*		-	*-*	38	−0.464	*0.0043*		-	*-*		-	*-*
IL-33	PV		-	*-*		-	*-*		-	*-*		-	*-*	24	0.499	*0.0154*
HGF	PV	24	0.501	*0.0149*		-	*-*		-	*-*		-	*-*		-	*-*
MIG	PV	24	0.462	*0.0266*		-	*-*		-	*-*	24	0.450	*0.0275*		-	*-*
TGF-β_1_	ET		-	*-*		-	*-*		-	*-*	38	−0.489	*0.0024*		-	*-*

–: not significant. Molecules shown in bold characters are those for which correlations with blood counts were found with a *r* > 0.600; Dg = diagnosis (MPN subtype); M = male patients only.

## Supplementary Materials

The following are available online at https://www.mdpi.com/2072-6694/12/9/2446/s1, Figure S1: Representation of the % of *JAK2*V617F-mutated alleles of the MPN patients examined in this study, Figure S2: Levels of ST-2 (soluble IL-33 receptor) in MPN patients, Figure S3: Inverse correlations between hematocrit and IL-2, IL-4 and IL-26 in ET patients, Figure S4: Description of UT-7 clones expressing 50% or 75% *JAK2*V617F-mutated alleles, Figure S5: Cytokine production of *CALR*-mutated UT-7 cells, Table S1: Correlations between %*JAK2*V617F and blood counts in *JAK2*V617F-mutated MPN patients, Table S2: Characteristics of ET patients with *JAK2*V617F or *CALR* mutation, Table S3: Characteristics of MPN patients with GlcSph-reactive IgGs.
