# Supplementary material for: Anti-Glucosylsphingosine Autoimmunity, JAK2V617F-Dependent Interleukin-1β and JAK2V617F-Independent Cytokines in Myeloproliferative Neoplasms
Source: Cancers (Basel). 2020 Aug 28;12(9):2446. doi: 10.3390/cancers12092446 (PMC7564615; doi:10.3390/cancers12092446)
Supplement: Supplementary file 1 [file cancers-12-02446-s001.pdf]

cancers MDPI

Article

Anti-Glucosylsphingosine Autoimmunity, JAK2V617F-Dependent Interleukin-1β and JAK2V617F-Independent Cytokines in Myeloproliferative Neoplasms

Sophie Allain-Maillet, Adrien Bosseboeuf, Nicolas Mennesson, Mégane Bostoën, Laura Dufeu, Eun Ho Choi, Cédric Cleyrat, Olivier Mansier, Eric Lippert, Yannick Le Bris, Jean-Marc Gombert, François Girodon, Magali Pettazzoni, Edith Bigot-Corbel and Sylvie Hermouet

Supplementary Materials:

Table S1. Correlations between %*JAK2*V617F and blood counts in *JAK2*V617F-mutated MPN patients.

Blood Parameter	All MPNs			PV			ET			PMF		
	n	r	*p value*	n	r	*p value*	n	r	*P value*	n	r	*p value*
Leukocytes (x10⁹/L)	53	0.4015	*0.0029*	25	0.7266	*<0.0001*	-	-	-	-	-	-
Neutrophils (x10⁹/L)	51	0.4321	*0.0015*	24	0.7885	*<0.0001*	-	-	-	-	-	-
Monocytes (x10⁹/L)	-	-	-	25	0.4018	*0.0465*	-	-	-	-	-	-
Hematocrit (L/L)	-	-	-	-	-	-	-	-	-	-	-	-
Hemoglobin (g/dL)	-	-	-	-	-	-	-	-	-	-	-	-
Platelets (x10⁹/L)	-	-	-	-	-	-	-	-	-	-	-	-

- : non-significant.

Table S2. Characteristics of ET patients with *JAK2*V617F or *CALR* mutation.

	*JAK2*V617F –Mutated ET	*CALR* –Mutated ET
**Number**	21	15
**Sex,** M/F (male%)	8/13 (38.1%)	7/8 (46.7%)
**Age** (Yr)		
Median	68.0	61.1
Range	(43.0–95.0)	(41.2–85.8)
**%*JAK2*V617F**		
Median	13.0	0
Range	(4.0–61.0)[a]	(0 –0)
**Blood Counts**		
Hematocrit (L/L)		
Median	46.2	42.9*
Range	(35.5–63.3)	(35.3–48.3)
Hemoglobin (g/dL)		

Median	14.8	14.0
Range	(10.4–20.0)	(11.6–11.6)
Leukocytes (x10⁹/L)		
Median	9.1	7.9
Range	(4. –41.0)	(5.3–15.4)
Platelets (x10⁹/L)		
Median	615	808**
Range	(191–1,851)	(360–2,300)

(a) Patient with myelofibrosis; *p = 0.0333 and **p = 0.0510 *vs* *JAK2*V617F-mutated ET, Mann-Whitney t-test.

Table S3. Characteristics of MPN patients with GlcSph-reactive IgGs.

	All Patients	PV	ET	PMF
**Patients with GlcSph–reactive IgGs**				
**Number**	15	3	8	4
**Sex,** M/F (male%)	6/9 (40.0%)	2/1 (66.7%)	2/6 (25.0%)	2/2 (50.0%)
**Age** (Yr)				
Median	67.0	67.0	67.4	64.0
(Range)	(33–95)	(33–76)	(41–95)	(50–73)
**%*JAK2*V617F**				
Median	16.0	25.0	0	27.0
(Range)	(0–96)	(17.0–96)	(0–34)	(0–37)
Blood Counts				
Hematocrit (L/L)				
Median	42.6	48.7	42.9	37.4
(Range)	(29.3–63.3)	(43.1–52.2)	(35.3–63.3)	(29.3–41.0)
Hemoglobin (g/dL)				
Median	14.0	14.1	14.0	11.6
(Range)	(9.6–20.0)	(13.8–16.7)	(11.6–20.0)	(9.6–13.0)
Leukocytes (x10⁹/L)				
Median	7.3	7.8	6.9	6.5
(Range)	(4.5–37.8)	(7.3–37.8)	(4.5–15.4)	(5.0–12.5)
Platelets (x10⁹/L)				
Median	483	349	765	116
(Range)	(47–2,300)	(239–493)	(191–2,300)	(47–483)
**Patients without GlcSph–reactive IgGs**				
**Number**	60	24	31	5
**Sex,** M/F (male%)	32/28 (53.3%)	14/10 (58.3%)	13/18 (41.9%)	5/0 (100%)
**Age** (Yr)				
Median	67.0	69.0	62.0	80.0
(Range)	(37–93)	(37–93)	(37–93)	(69–86)
**%*JAK2*V617F**				
Median	15.5	17.6	14.4	10.5
(Range)	(7.9–22.4)	(15.5–22.4)	(10.4–16.2)	(7.9–12.1)
Blood Counts				

Hematocrit (L/L)				
Median	47.2	55.2	44.4	33.7
(Range)	(25.1–70.0)	(48.2–70.0)	(35.5–51.8)	(25.1–38.8)
Hemoglobin (g/dL)				
Median	15.5	17.6	14.4	10.5
(Range)	(7.9–22.4)	(15.5–22.4)	(10.4–16.2)	(7.9–12.1)
Leukocytes (x10⁹/L)				
Median	9.9	9.8	8.7	17.3
(Range)	(2.8–41.0)	(2.8–24.6)	(5.3–41.0)	(10.2–27.5)
Platelets (x10⁹/L)				
Median	540	383	634	239
(Range)	(76–2,160)	(89–851)	(360–2,160)	(76–586)

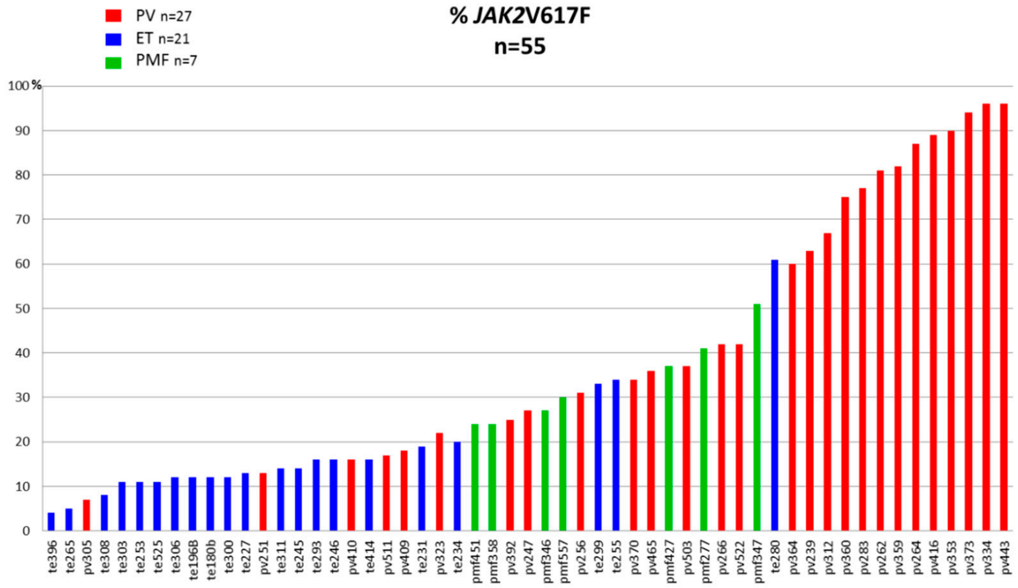

Figure S1. Representation of the % of *JAK2*V617F-mutated alleles of the MPN patients examined in this study.

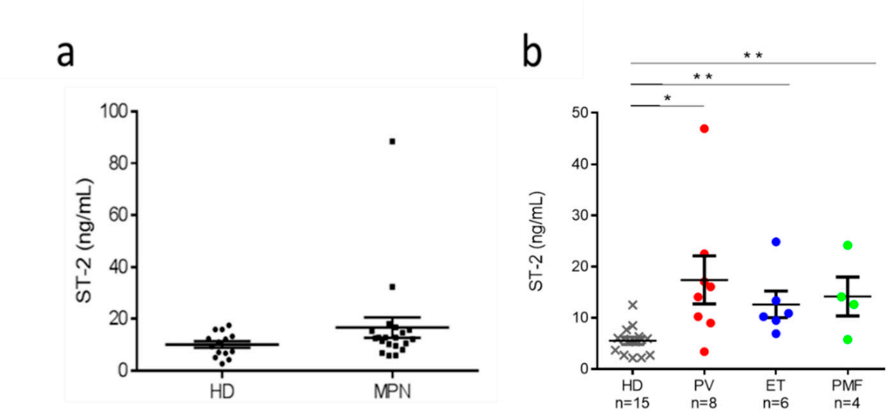

Figure 2. Levels of ST-2 (soluble IL-33 receptor) in MPN patients. ST-2 was measured in parallel in serum (a) and in plasma (b) of MPN patients and healthy donors (HD), as previously described (Levescot et al. 2014; Mager et al. 2018). Data are presented as means ± SEM. (*) $p < 0.05$, (**) $p < 0.01$, Mann-Whitney *t*-test. Note the change in scale between (a) and (b).

References

Levescot, A.; Flamant, S.; Basbous, S.; Jacomet, F.; Féraud, O.; Bourgeois, E.A.; Bonnet, M.-L.; Giraud, C.; Roy, L.; Barra, A.; Chomel, J.-C.; Turhan, A.; Guilhot, F.; Girard, J.-P.; Gombert, J.-M.; Herbelin, A. BCR-ABL-induced Deregulation of the IL-33/ST2 Pathway in CD34+ Progenitors From Chronic Myeloid Leukemia Patien**ts.** *Cancer Res* **2014**, *74*, 2669–2676.

Mager, B.L.F.; Riether, C.; Schurch, C.M.; et al. IL-33 signalling contributes to the pathogenesis of myeloproliferative neoplasms. *J Clin Invest* **2015**, *125*, 2579–2591.

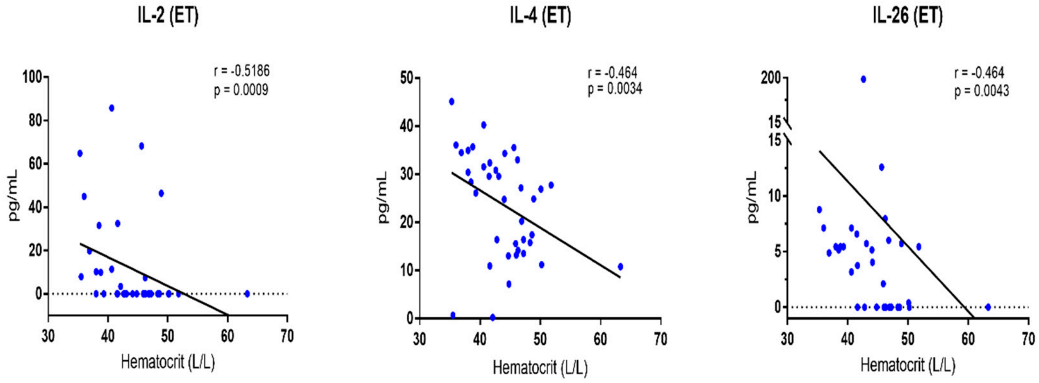

Figure S3. Inverse correlations between hematocrit and IL-2, IL-4 and IL-26 in ET patients. Analysis of the cytokine levels in serum and blood parameters in ET patients, using Spearman's *t*-test, revealed negative correlations between hematocrit and the levels of IL-2, IL-4, and IL-26.

a

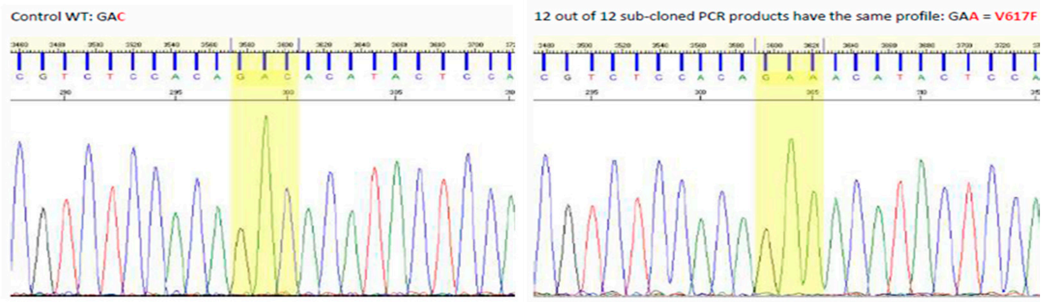

b

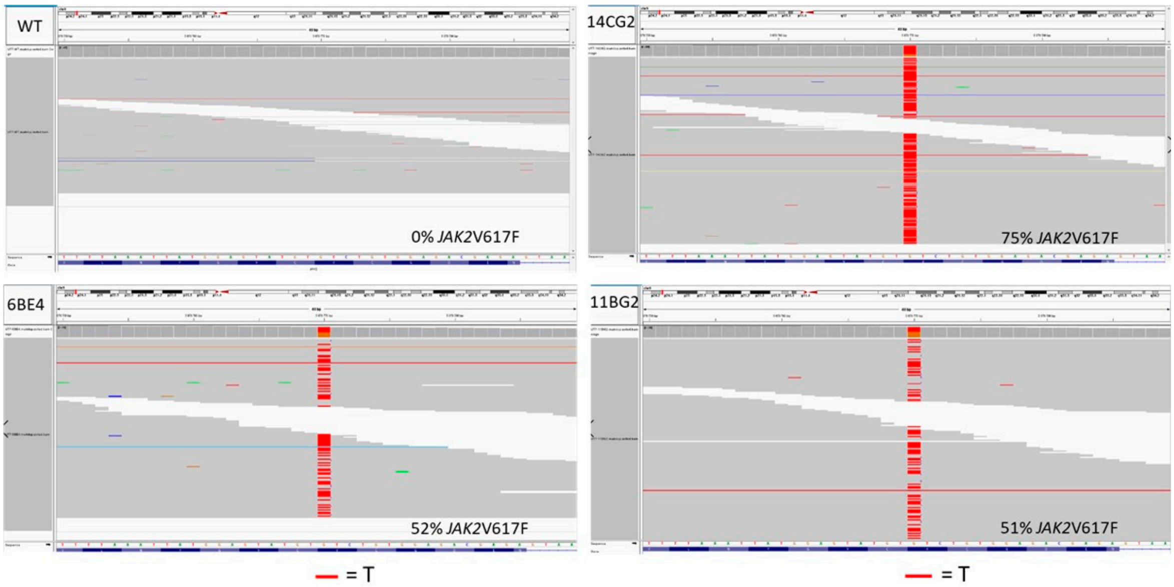

c

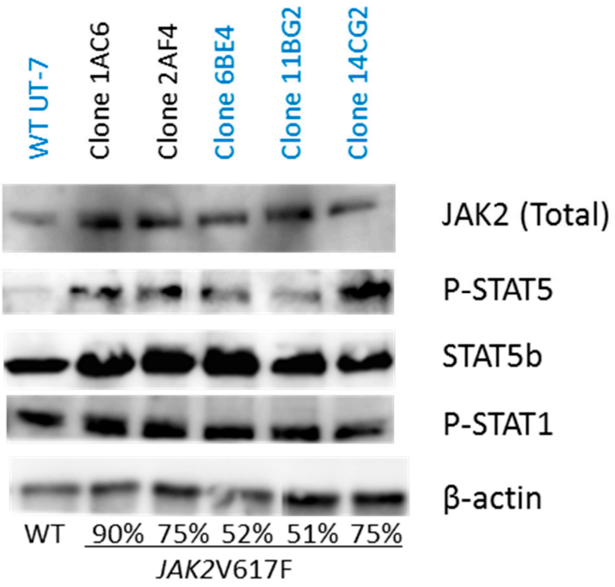

Figure S4. Description of UT-7 clones expressing 50% or 75% *JAK2*V617F-mutated alleles. (**a**) Sanger sequencing: Reverse sequencing of wild-type (WT) *JAK2* UT-7 cells (left panel) showed the expected GAC sequence of WT *JAK2* (sense sequence: GTC) whereas *JAK2*V617F-mutated UT-7 cells showed the GAA sequence corresponding to *JAK2*V617F (sense sequence: TTC) (right panel). (**b**)Whole genome sequencing: Results of *JAK2* sequencing obtained using the Integrative Genomics Viewer (IGV) (http://software.broadinstitute.org/software/igv/). Alleles with a sequence identical to the *JAK2* reference sequence (GTC) appear in grey (WT UT-7 cells), whereas red sequence reads correspond to a T mutation (TTC). The proportion of red reads is similar for UT-7 clones 6BE4 and 11BG2, and higher for clone 14CG2. The % of *JAK2*V617F-mutated alleles, quantified using allele specific qPCRs, were 52% for the 6BE4 UT-7 clone, 51% for the 11BG2 UT-7 clone, and 75% for the 14CG2 UT-7 clone. (**c**) JAK2/STAT5 pathway in UT-7 clones expressing 50% or 75% *JAK2*V617F-mutated alleles: WT *JAK2* UT-7 cells and the 3 UT-7 clones used in this study (shown in blue, expressing 51%, 52% or 75% *JAK2*V617F-mutated alleles) were grown in the presence of GM-CSF, then washed three times, numerated, seeded at 10^5 cells/ml in MEM with 10% FCS and a reduced dose of GM-CSF (1 ng/ml), and incubated at 37°C for 18 hrs. Cells were then collected, washed, and cell pellets were frozen at -80°C until use. Cell lysates were prepared and submitted to SDS-PAGE and transfer to PDVF

membranes, and blots were incubated with anti-human JAK2, STAT5b and β-actin antibodies or with anti-human phospho (P)-JAK2, P-STAT5 and P-STAT1 antibodies, then blots were revealed by chemiluminescence, as published (Cleyrat *et al.* 2010; Boissinot *et al.* 2011). JAK2V617F-expressing UT-7 clones showed higher expression of total JAK2 and increased basal tyrosine-phosphorylation of STAT5. Expression of STAT5b was similar in WT UT-7 cells and in *JAK2*V617F-mutated UT-7 clones. There was no increase in STAT1 tyrosine-phosphorylation in *JAK2*V617F-mutated UT-7 clones.

References

Cleyrat, C.; Jelinek, J.; Girodon, F.; Boissinot, M.; Ponge, T.; Harousseau, J.-L.; Issa, J.-P.; Hermouet, S. JAK2 mutation and disease phenotype: A double L611V/ V617F *in cis* mutation of JAK2 is associated with isolated erythrocytosis and increased activation of AKT and ERK1/2 rather than STAT5. *Leukemia* **2010**, *24*, 1069–1073.

Boissinot, M.; Cleyrat, C.; Vilaine, M.; Jacques, Y.; Corre, I,.; Hermouet S. Anti-inflammatory hepatocyte growth factor and interleukin-11 are overexpressed in Polycythemia Vera and contribute to the growth of mutated erythroblasts independently of *JAK2*V617F. *Oncogene* **2011**, *30*, 990–1001.

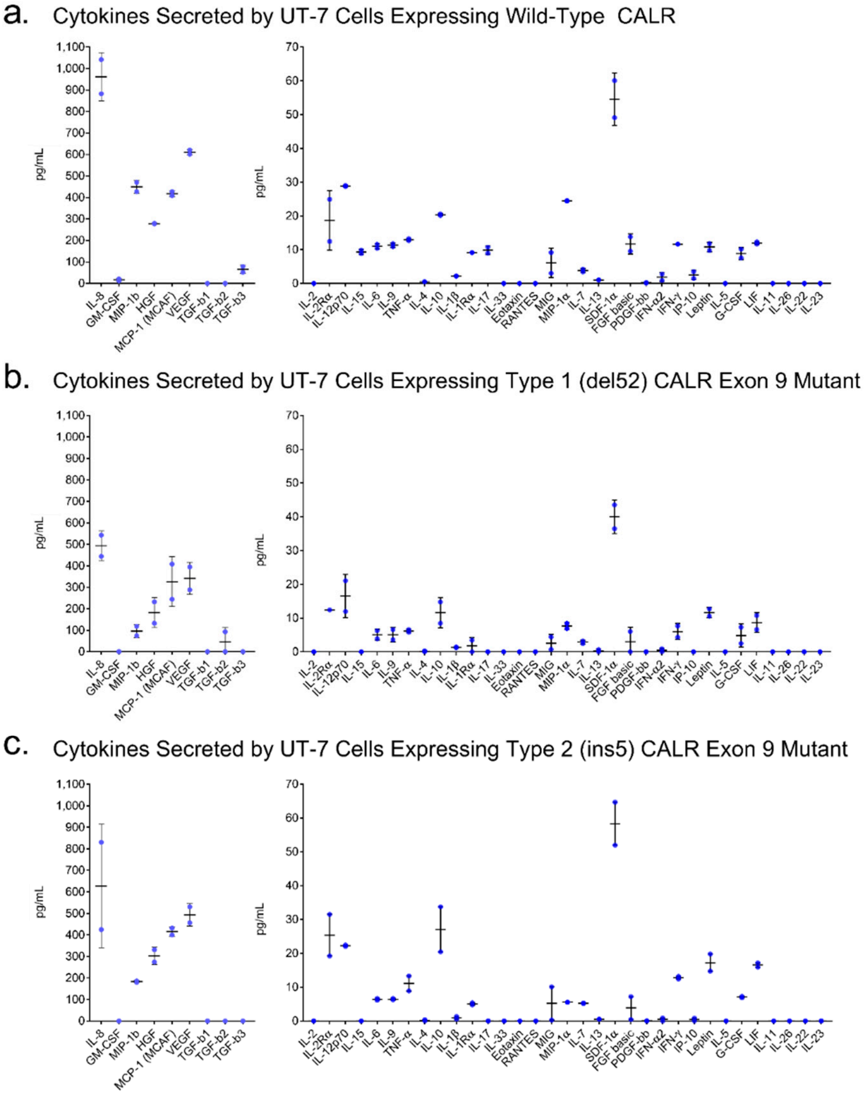

Figure S5. Cytokine production of *CALR*-mutated UT-7 cells. The basal level of 40 cytokines and chemokines and 2 soluble receptors were quantified in the supernatants of (a) wild-type *CALR* UT-7 cells; (b) UT-7 cells expressing type 1 (del52) *CALR* exon 9 mutant; (c) UT-7 cells expressing type 2 (ins5) *CALR* exon 9 mutant. Note that 9 cytokines were produced at high levels (left panels). Results are presented as means ± SEM and horizontal black bars represent the mean values.

© 2020 by the authors. Submitted for possible open access publication under the terms and conditions of the Creative Commons Attribution (CC BY) license (http://creativecommons.org/licenses/by/4.0/).
